# Mononuclear Phagocytes and Airway Epithelial Cells: Novel Sources of Matrix Metalloproteinase-8 (MMP-8) in Patients with Idiopathic Pulmonary Fibrosis

**DOI:** 10.1371/journal.pone.0097485

**Published:** 2014-05-14

**Authors:** Vanessa J. Craig, Francesca Polverino, Maria E. Laucho-Contreras, Yuanyuan Shi, Yushi Liu, Juan C. Osorio, Yohannes Tesfaigzi, Victor Pinto-Plata, Bernadette R. Gochuico, Ivan O. Rosas, Caroline A. Owen

**Affiliations:** 1 Division of Pulmonary and Critical Care Medicine, Brigham and Women's Hospital, Harvard Medical School, Boston, Massachusetts, United States of America; 2 Department of Clinical and Experimental Medicine, University of Parma, Parma, Italy; 3 Pulmonary Fibrosis Program, Lovelace Respiratory Research Institute, Albuquerque, New Mexico, United States of America; 4 Chronic Obstructive Pulmonary Disease Program, Lovelace Respiratory Research Institute, Albuquerque, New Mexico, United States of America; 5 Medical Genetics Branch, National Human Genome Research Institute, National Institutes of Health, Bethesda, Maryland, United States of America; University of Pittsburgh, United States of America

## Abstract

**Objectives:**

Matrix metalloproteinase-8 (MMP-8) promotes lung fibrotic responses to bleomycin in mice. Although prior studies reported that MMP-8 levels are increased in plasma and bronchoalveolar lavage fluid (BALF) samples from IPF patients, neither the bioactive forms nor the cellular sources of MMP-8 in idiopathic pulmonary fibrosis (IPF) patients have been identified. It is not known whether MMP-8 expression is dys-regulated in IPF leukocytes or whether MMP-8 plasma levels correlate with IPF outcomes. Our goal was to address these knowledge gaps.

**Methods:**

We measured MMP-8 levels and forms in blood and lung samples from IPF patients versus controls using ELISAs, western blotting, and qPCR, and assessed whether MMP-8 plasma levels in 73 IPF patients correlate with rate of lung function decline and mortality. We used immunostaining to localize MMP-8 expression in IPF lungs. We quantified MMP-8 levels and forms in blood leukocytes from IPF patients versus controls.

**Results:**

IPF patients have increased BALF, whole lung, and plasma levels of soluble MMP-8 protein. Active MMP-8 is the main form elevated in IPF lungs. MMP-8 mRNA levels are increased in monocytes from IPF patients, but IPF patients and controls have similar levels of MMP-8 in PMNs. Surprisingly, macrophages and airway epithelial cells are the main cells expressing MMP-8 in IPF lungs. Plasma and BALF MMP-8 levels do not correlate with decline in lung function and/or mortality in IPF patients.

**Conclusion:**

Blood and lung MMP-8 levels are increased in IPF patients. Active MMP-8 is the main form elevated in IPF lungs. Surprisingly, blood monocytes, lung macrophages, and airway epithelial cells are the main cells in which MMP-8 is upregulated in IPF patients. Plasma and BALF MMP-8 levels are unlikely to serve as a prognostic biomarker for IPF patients. These results provide new information about the expression patterns of MMP-8 in IPF patients.

## Introduction

IPF is associated with high morbidity and mortality [1] and its incidence is increasing [2], [3]. No therapies have been shown to reduce mortality in IPF patients [4]–[6]. IPF is thought to be caused by an initial (as-yet unidentified) alveolar epithelial injury which is followed by an aberrant wound healing response in the lung [7], [8], but the pathways that contribute to this response are not fully understood. An improved understanding of the mechanisms involved in the pathogenesis of IPF may facilitate the development of more efficacious therapeutic approaches for this disease.

One molecule that has recently been linked to fibrotic lung diseases is matrix metalloproteinase-8 (MMP-8 or neutrophil collagenase). MMP-8 degrades type I collagen (the major collagen deposited in IPF lungs) in vitro [9]. However, recent studies have shown that MMP-8 promotes (rather than inhibits) lung fibrosis in bleomycin-treated mice, and this is linked to MMP-8's activities in reducing lung levels of macrophage inflammatory protein 1α (MIP-1α) and interferon-inducible protein-10 (IP-10) which is an anti-fibrotic cytokine [10]–[12].

MMP-8 is most highly expressed by neutrophils [13]. However, macrophages stimulated ex vivo with CD40 ligand [14] and activated fibroblasts [10], [15], [16] also express MMP-8 albeit at lower levels than neutrophils. All MMP-8-expressing cells release a soluble form of MMP-8 as a latent (pro)-proteinase which is then activated in the extracellular space. Activated neutrophils also express another form of MMP-8 on their surface in an inducible fashion [12], [17]. Membrane-bound MMP-8 on PMNs is catalytically active but resistant to inhibition by tissue inhibitors of metalloproteinases and could be the key form mediating its activities *in vivo* [12], [17]. However, membrane-bound MMP-8 on PMNs has not been studied in any human disease.

A small number of prior studies have linked MMP-8 to human IPF. One study reported that MMP-8 plasma levels are elevated in IPF patients [18], but this study did not assess whether plasma MMP-8 levels correlate with clinical outcomes or parameters in IPF patients. One study reported that MMP-8 expression was not increased in IPF whole lung samples [19]. Several other studies showed that IPF patients have elevated levels of soluble MMP-8 in bronchoalveolar lavage fluid (BALF) [18], [20]–[22]. One of these studies showed, in a small cohort of IPF subjects, that elevated MMP-8 BALF levels are associated with rapidly declining lung function [21].

Knowledge gaps in the area of MMP-8 and IPF include: 1) which cells in the lung contribute to the elevated BALF MMP-8 levels in IPF patients; 2) whether MMP-8 levels in blood myeloid leukocytes are dysregulated in IPF patients; 3) which is the key form of the proteinase (soluble vs. membrane bound and active vs. latent MMP-8) present in IPF blood and lung samples; and 4) whether substrates that we have identified for MMP-8 in the fibrotic murine lung are also potential substrates for MMP-8 in human IPF lungs. To address these knowledge gaps, we performed a comprehensive analysis of MMP-8 levels and forms in both blood and lung samples from IPF patients versus control subjects. We measured levels of substrates that we have identified for MMP-8 in the fibrotic murine lung (MIP-1α and IP-10) in IPF lung samples to begin to assess whether they might be substrates for MMP-8 in IPF lungs. We also assessed whether plasma MMP-8 levels can serve as a prognostic biomarker for IPF.

Based upon current knowledge of MMP-8 expression patterns, we hypothesized that MMP-8 is mainly expressed by neutrophils and fibroblasts in IPF lungs. As there is evidence that blood neutrophils are activated and undergo degranulation in IPF patients [23], we also hypothesized that MMP-8 levels would be lower in extracts of blood neutrophils and/or higher on the surface of blood neutrophils from IPF patients compared with controls. However, we report for the first time that lung macrophages and airway epithelial cells are the key sources of MMP-8 in IPF lungs. Although we confirmed that plasma MMP-8 levels are increased in IPF patients, surprisingly, MMP-8 levels are not altered in neutrophils from IPF patients. Rather, IPF patients have increased expression of MMP-8 in blood monocytes. Thus, we provide new information about expression and activation patterns of MMP-8 in IPF lung samples which may guide future biomarker studies, and possibly the testing of novel therapeutics targeting MMP-8 for IPF.

## Materials and Methods

### Human subjects

All research involving human participants was approved by the authors' institutional review board [The Partners Health Care Institutional Review Board (IRB) under protocols #2011P002419 and 2002P000253]. All study subjects signed written informed consent forms that were approved by our IRB. All clinical investigations were conducted according to the principles expressed in the Declaration of Helsinki.

Blood or BALF samples were obtained from healthy volunteers (n = 25 or 12, respectively) and IPF patients (n = 73 or 32, respectively) enrolled in the Brigham and Women's Hospital Interstitial Lung Disease Registry or who signed informed consent and were enrolled in NIH protocols (99-HG-0056 and 04-HG-0211). The diagnosis of IPF was made using ATS/ERS consensus diagnostic criteria [24]. Forced vital capacity (FVC) and diffusing capacity of the lung for carbon monoxide (DLCO)] were recorded on IPF patients for clinical indications. Explanted lung samples from IPF patients undergoing lung transplantation and controls (rejected donor lung transplant tissue) were randomly selected from our IPF bio-repository.

### MMP-8, MIP-1α, and IP-10 levels

MMP-8 was quantified in blood and lung samples using an ELISA (R&D Systems, Minneapolis, MN). MMP-8 levels in homogenates of lung samples were corrected for GAPDH levels which were measured in arbitrary units using a commercial kit (eBioscience, San Diego, CA). MMP-8 results were expressed as pg of MMP-8 per arbitrary unit of GAPDH. MIP-1α and IP-10 were quantified in BALF samples using ELISAs (PeproTech, Rocky Hill, NJ). MMP-8 forms were analyzed in BALF (50 microliters/sample) and lung lysates (100 micrograms of protein/sample) using western blotting [12], [17] and a polyclonal rabbit anti-human MMP-8 IgG [ab38994; raised against the hinge region of MMP-8 (Abcam, Cambridge, MA)] and quantified using ImageJ software [25].

### Leukocyte studies

Neutrophils and monocytes were isolated from blood using density gradient centrifugation [26] and positive selection for CD14 using immuno-magnetic beads (Miltenyi Biotec, San Diego, CA), respectively. Cells were lysed in radio-immunoprecipitation assay (RIPA) buffer containing protease inhibitors (at 5×10^6^ cells/ml), and frozen at −80°C. Intact neutrophils were immunostained for surface MMP-8 using Alexa 488 and rabbit anti-MMP-8 IgG (ab38994, Abcam) or non-immune rabbit IgG as a control (Dako, Carpinteria, CA) [12], [17] and staining quantified using a FACS Canto II flow cytometer (BD, Franklin Lakes, NJ).

### Real-time RT-PCR

Real-time RT-PCR was performed on RNA isolated from blood leukocytes and lungs using a MMP-8 gene expression assay (Invitrogen, Eugene, OR), and the comparative cycle threshold method with 18S as an endogenous reference gene [10].

### Immunoperoxidase staining

Formalin-fixed lung sections from IPF patients and control subjects were deparaffinized. Antigen retrieval was performed by boiling the sections in 10 mM citrate buffer (pH 6.0) in a microwave for 10 min. Slides were incubated in blocking buffer [1% (w/v) BSA and 10% (v/v) goat serum in Tris buffered saline (TBS; 0.05M Tris containing 0.15 M NaCl and 0.02 M CaCl_2_] for 2 h at room temperature. Slides were then incubated with either rabbit anti-MMP-8 IgG or non-immune rabbit IgG for 18 h at 4°C and washed twice in TBS. Slides were incubated in 3% hydrogen peroxide solution for 20 min, washed, incubated again with hydrogen peroxide solution, washed, and incubated for 1 h at room temperature with goat anti-rabbit IgG conjugated to horseradish peroxidase (Bio-Rad, Berkeley, California). Slides were washed, incubated in avidin-biotin complex for 1 h at room temperature, washed again, and developed using 3,3′-diaminobenzidine. Slides were then counterstained with 1% (wt/vol) methyl green solution, dehydrated, and mounted.

### Immunofluorescence staining to localize MMP-8 in lung sections

Formalin-fixed lung sections from IPF patients and controls were deparaffinized, and antigen retrieval was performed by heating the slides in a microwave in citrate buffer, as outlined above. The sections were incubated overnight at 4°C with rabbit IgG to human MMP-8 (or non-immune rabbit IgG) and Alexa 546-conjugated goat anti-rabbit F(ab)_2_. Sections were then washed in PBS and incubated at 37°C for 2 h with either murine anti-CD68 IgG (Dako, Carpinteria, CA), murine anti-vimentin IgG (Abcam), murine anti-surfactant protein C IgG (SP-C, Santa Cruz Biotechnology, Santa Cruz, CA.), murine anti-pancytokeratin IgG (Sigma-Aldrich, St. Louis, MO), or non-immune murine IgG [27]. After washing the lung sections in PBS, Alexa 488-conjugated goat anti-murine F(ab)_2_ was applied and slides were incubated for additional 1 h at 37°C. Nuclei were then counterstained with 4′,6-diamidino-2-phenylindole (DAPI).

### Immunostaining to detect apoptosis of alveolar type II epithelial cells in lung sections

To detect the alveolar epithelial type II (ATII) cells undergoing apoptosis, we performed double immunofluorescence staining of lung sections for active (cleaved) caspase-3 and a marker of ATII cells (surfactant protein C; SP-C). Formalin-fixed lung sections from IPF patients and controls were deparaffinized, and antigen retrieval was performed by heating the slides in a microwave in citrate buffer. The sections were incubated overnight at 4°C with murine anti-active caspase-3 IgG (Abcam, Cambridge, MA), or non-immune murine IgG (Sigma-Aldrich, St. Louis MO) followed by Alexa 488-conjugated goat anti-murine F(ab)_2_. Slides were washed in PBS and incubated with goat anti-SP-C IgG (Santa Cruz Biotechnology, Santa Cruz, CA.) or non-immune goat IgG (Sigma-Aldrich, St. Louis, MO) followed Alexa 546-conjugated rabbit anti-goat F(ab)_2_. Nuclei were counterstained with DAPI.

### Statistics

The results for paired and unpaired data were compared using the Student's t-test for parametric data and the Mann-Whitney rank sum test for non-parametric data; P values less than 0.05 were considered significant. To study associations between MMP-8 plasma levels and FVC or DLCO and, results from patients with ≥2 clinic visits were analyzed and FVC and DLCO results from the second and third visits were expressed as a % of baseline values. Linear regression was performed for each patient and the slope was used to calculate the annual percent change in relative and absolute FVC and DLCO, and Spearman Rank Correlation Coefficients for non-parametric data were calculated. The Cox proportional-hazards regression model was used to determine whether there is a relationship between plasma MMP-8 levels and survival in IPF patients.

## Results

### MMP-8 levels in IPF lungs

**Figure 1:**
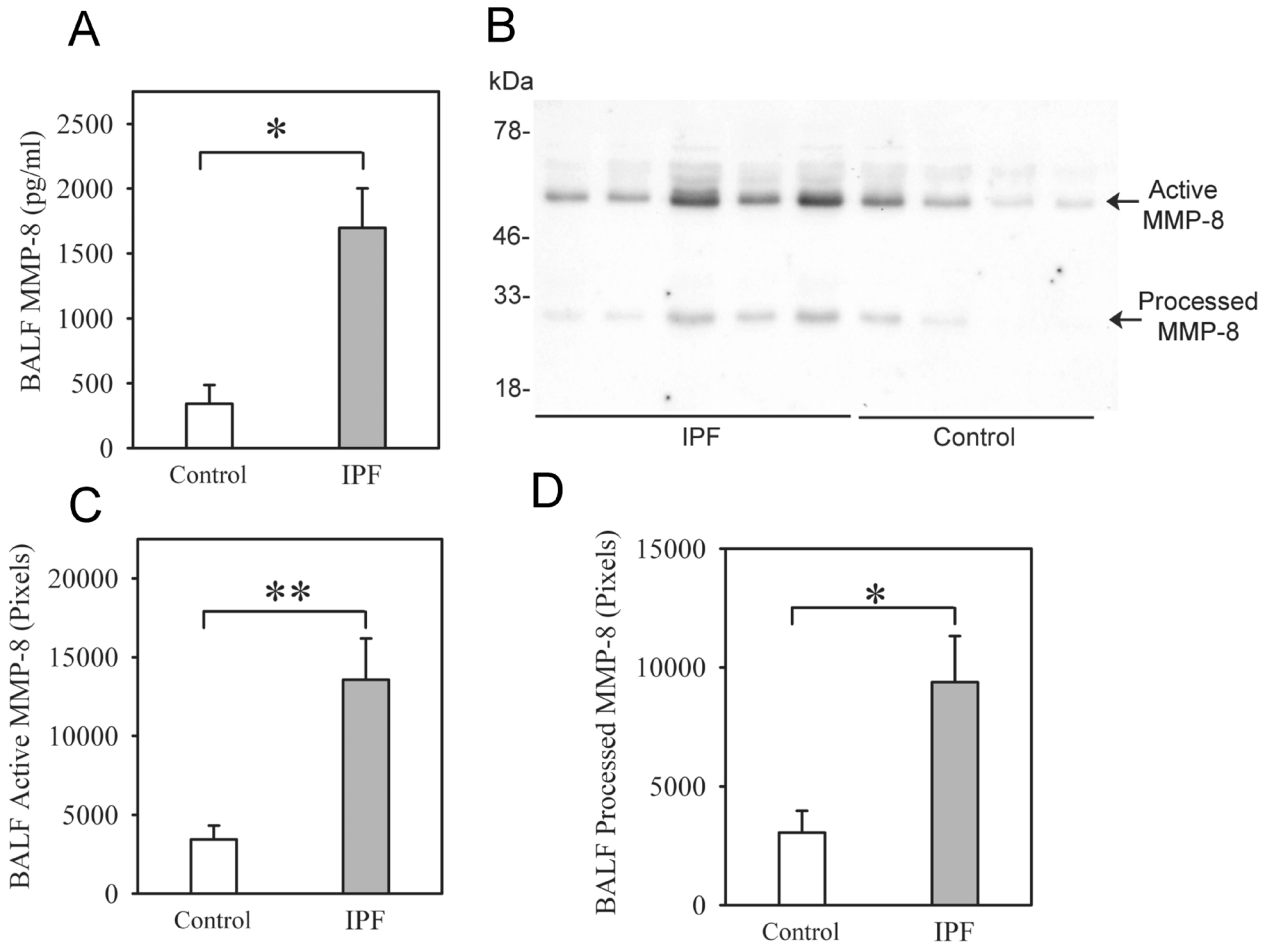
MMP-8 protein levels are increased in IPF BALF samples. In **A**, BAL was performed on IPF patients (n = 32) and healthy volunteers (n = 7), and MMP-8 protein levels were quantified in BAL fluid (BALF) samples using an ELISA. Data are mean + SEM; * indicates p = 0.003. In **B**, we analyzed BALF samples from both groups using Western blotting, and detected both active MMP-8 protein (∼60 kDa) and processed MMP-8 (∼40 kDa) forms in BALF from IPF patients and healthy volunteers. **B** shows a representative blot of BALF samples from 5 IPF patients and 4 control subjects. In **C**–**D**, densitometry was used to quantify levels of active MMP-8 (in **C**) and processed MMP-8 (in **D**) in BALF samples. In **C** and **D**, data are mean + SEM; n = 9 control samples and n = 9 IPF samples; asterisk indicates p = 0.022 and ** p = 0.002.

**Figure 2:**
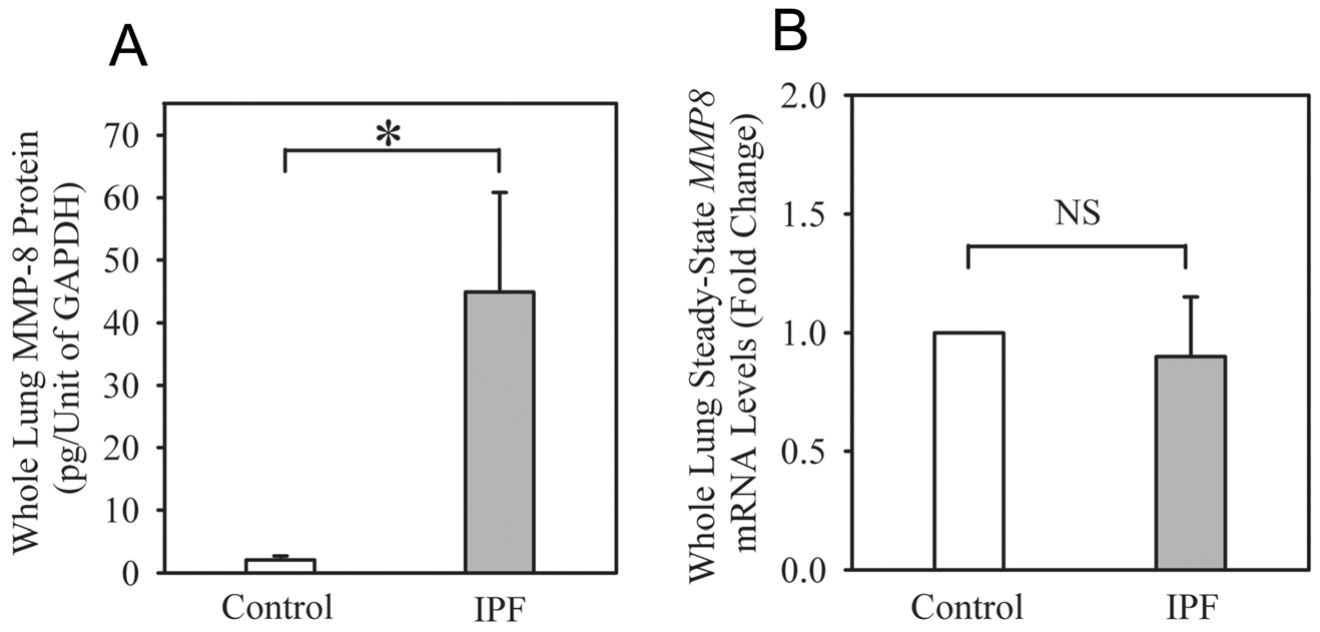
MMP-8 protein levels are increased in IPF whole lung samples. Lung tissue was removed from explanted lungs from IPF patients during lung transplantation (n = 5) or unused transplant donor lungs (control; n = 5). In **A**, MMP-8 protein levels were measured in homogenates of lung samples using an ELISA. Results for MMP-8 were normalized to GAPDH levels (expressed as pg of MMP-8 per arbitrary unit of GAPDH). Data are mean + SEM. Asterisk indicates p<0.03. In **B**, MMP-8 steady-state mRNA levels were measured in RNA isolated from whole lung samples. Data are fold change + SEM; n = 5 control subjects and 4 IPF patients.

Soluble MMP-8 protein levels are ∼5-fold higher in BALF from IPF patients compared with healthy volunteers (Fig. 1A and Table S1 for demographic data on the subjects). Two main forms of MMP-8 are detected in BALF using Western blotting (Fig. 1B): 1) active MMP-8 (M_r_∼60 kDa) which is the most abundant form; and 2) a ∼40 kDa form which likely is a proteolytically processed and inactive form of MMP-8 (containing its hinge region and the hemopexin MMP-8 domains) which we have detected in BALF from mice with acute lung injury [12]. Both forms are increased in IPF BALF (Figs. 1C and 1D). Latent pro-MMP-8 (M_r_∼80 kDa) is not detected or present at only low levels in BALF samples from IPF cases and controls. MMP-8 protein levels are also strikingly increased in homogenates of lung samples from IPF patients (Fig. 2A), but whole lung MMP-8 steady-state mRNA levels are similar in IPF patients and control subjects (Fig. 2B).

### MMP-8 localization in IPF lungs

**Figure 3:**
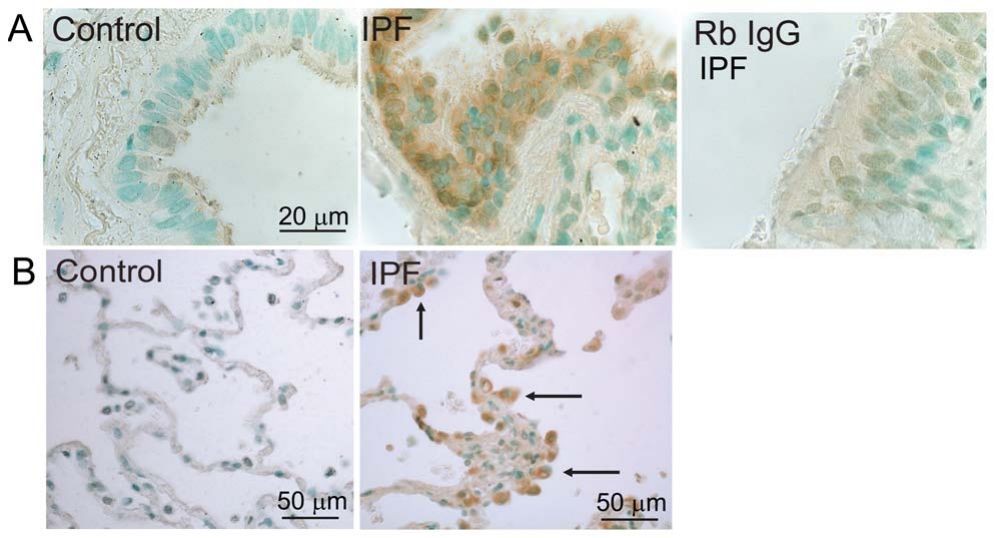
Immunostaining for MMP-8 is increased in IPF lungs. Lung tissue was obtained from IPF patients undergoing lung transplantation and unused transplant donor lungs (control). Immunoperoxidase staining for MMP-8 was performed on formalin-fixed IPF and control lung sections. In **A**, there is positive (brown) staining for MMP-8 in bronchial epithelial cells in an area of severe fibrosis in the IPF lung but not in bronchial epithelial cells in the control lung section. **B** shows positive (brown) staining for MMP-8 in cells in fibrotic parenchyma in IPF lungs (indicated by the arrows) but not in control lung parenchyma. Lung sections stained with a non-immune rabbit IgG control primary antibody (Rb IgG) showed minimal staining. These results are representative of 3 different lung sections per group. Magnification is X 400.

Based upon current knowledge of MMP-8 expression patterns, we hypothesized that MMP-8 is mainly expressed by PMNs and fibroblasts in IPF lungs. To test this hypothesis, we first performed immunoperoxidase staining of lung sections for MMP-8. There is robust staining for MMP-8 in bronchial epithelial cells in areas of moderately severe and severe fibrosis in IPF lungs. However, there is minimal or no staining for MMP-8 in areas of mild fibrosis in IPF lung and in rejected normal lung transplant donor lungs (Fig. 3A). In addition, positive staining for MMP-8 is present in cells in fibrotic lung tissue but not in control lung tissue (Fig. 3B), and no staining in IPF lung stained with a non-immune control primary antibody (Fig. 3A, right panel).

**Figure 4:**
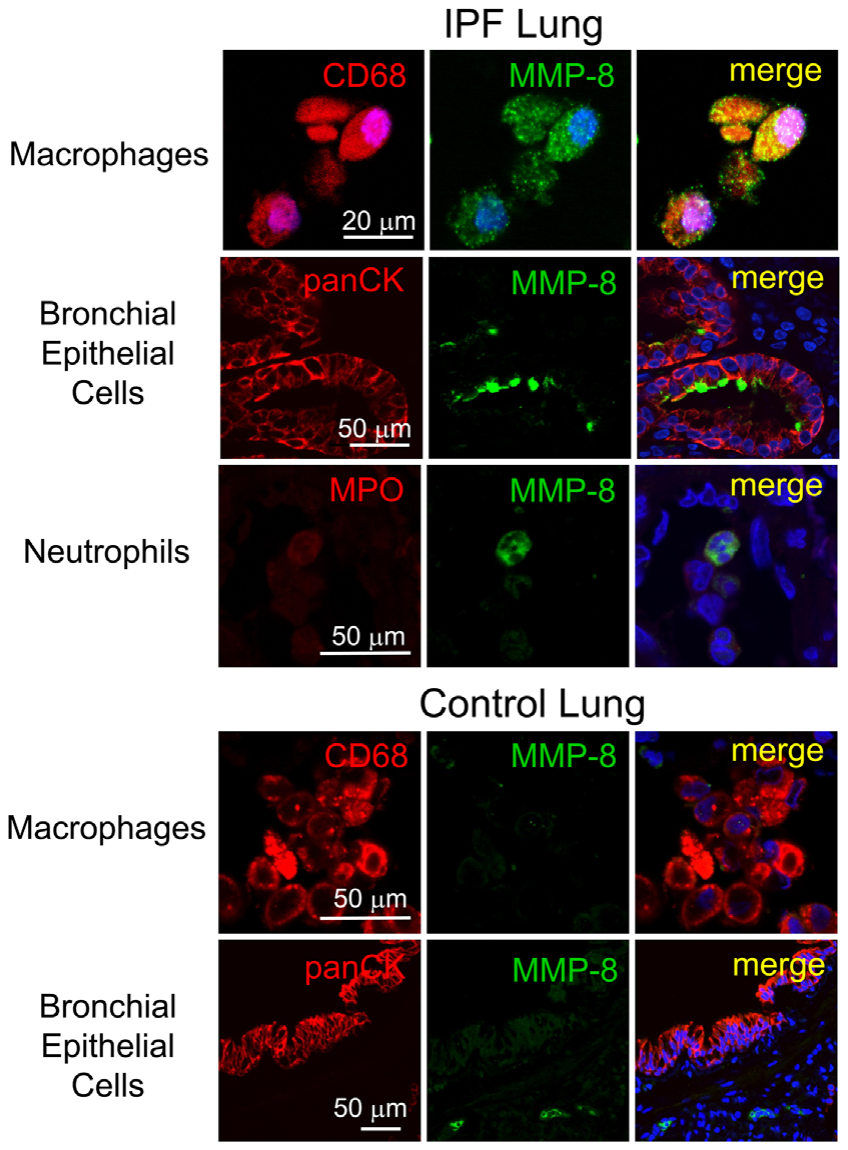
MMP-8 expression is increased in macrophages and bronchial epithelial cells in IPF lungs. Double immunofluorescence staining of an IPF lung section (upper panels) and a control lung section (lower panels) was performed using a red fluorophore (left column) for macrophages (CD68), airway epithelial cells (pancytokeratin; PanCK), or neutrophils (myeloperoxidase; MPO) and with a green fluorophore for MMP-8 (middle column). Lung sections were also stained with isotype-matched non-immune murine and rabbit IgG control antibodies (see Fig. 5). Nuclei were stained with 4′,6-diamidino-2-phenylindole (DAPI), and lung sections were examined using a confocal microscope. Merged images (right column) show co-localization of staining for MMP-8 and CD68 and also for MMP-8 and PanCK in the bronchial epithelium of an area of severe fibrosis in the IPF lung (upper panels). The control lung section (lower panels) shows no staining for MMP-8 in macrophages and minimal staining for MMP-8 (middle column) in bronchial epithelial cells. Images are representative of immuno-stained lung sections from 2 controls and 3 patients with IPF. Magnification is X 600 in both panels.

**Figure 5:**
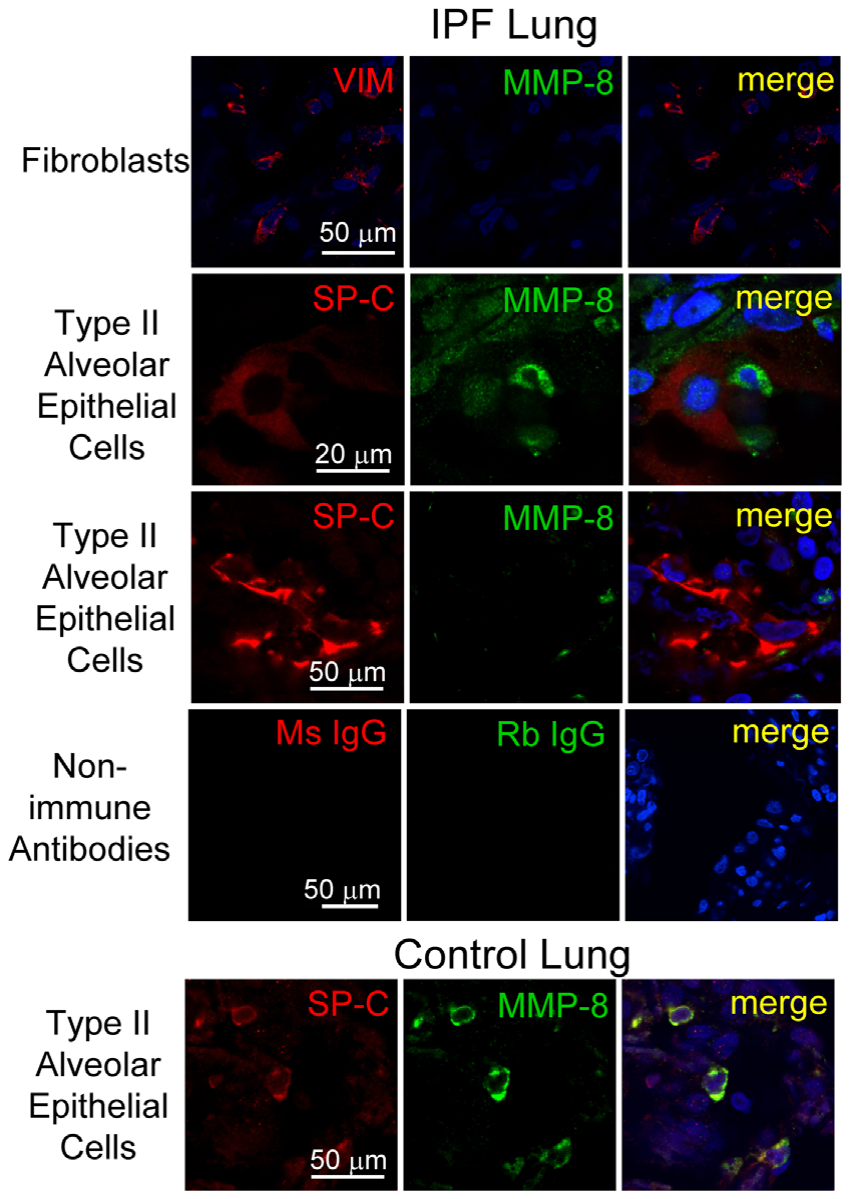
MMP-8 is not expressed in type II alveolar epithelial cells or fibroblasts in IPF lungs. Lung tissue was obtained from IPF patients undergoing lung transplantation (upper panel) or from rejected transplant donor lungs as a control (lower panel). Formalin-fixed lung sections were stained with a red fluorophore (left column) for fibroblasts (vimentin; VIM) or type II alveolar epithelial cells (surfactant protein C, SPC) and a green fluorophore for MMP-8 (middle column). Other lung sections were stained with isotype-matched non-immune murine IgG (Ms IgG) or rabbit IgG (Rb IgG). Nuclei were stained with DAPI and lung sections examined using a confocal microscope. Merged images (right column) show no staining for MMP-8 in cells identified as fibroblasts or type II alveolar epithelial (ATII) cells in an area of severe fibrosis in the IPF lung. Control lung sections show positive staining for MMP-8 in type II alveolar epithelial cells (lower panels). Magnification is X 600.

To confirm these results and identify the cell types in which MMP-8 is regulated in IPF lungs, we double immuno-stained lung sections from explanted IPF lungs and rejected normal lung transplant donor lungs using a green fluorophore for MMP-8 and a red fluorophore for markers of epithelial cells, macrophages, neutrophils, or fibroblasts. Macrophages are strongly stained for MMP-8 in all areas of the lung including areas of mild and severe fibrosis in IPF lung tissue (Fig. 4 upper panel). However, macrophages are not stained for MMP-8 in control lung samples (Fig. 4 lower panel). Bronchial epithelial cells in IPF lung tissue (Fig. 4, upper panel) are positively stained for MMP-8 mainly in areas of severe lung fibrosis (Fig. 4 upper panel). However, in control lung tissue, bronchial epithelial cells stain only weakly for MMP-8 (Fig. 4, lower panel). The few neutrophils present in IPF lungs stain strongly for MMP-8 (Fig. 4 upper panel). Fibroblasts (Fig. 5, upper panel) and fibrobastic foci (not shown) in IPF lung are not stained for MMP-8. Type II alveolar epithelial (ATII) cells stain strongly for MMP-8 in control lung tissue (Fig. 5, lower panel) but there is minimal or no staining for MMP-8 in these cells in areas of moderately severe and severe fibrosis in IPF lungs (Fig. 5). Staining for MMP-8 in ATII cells in areas of mild fibrosis in IPF lung is similar to that in normal lungs (data not shown). There is no staining in lung sections from IPF patients (Fig. 5) or control subjects (data not shown) incubated with isotype-matched non-immune control antibodies, confirming that our staining for MMP-8 and markers of different cell types is specific.

**Figure 6:**
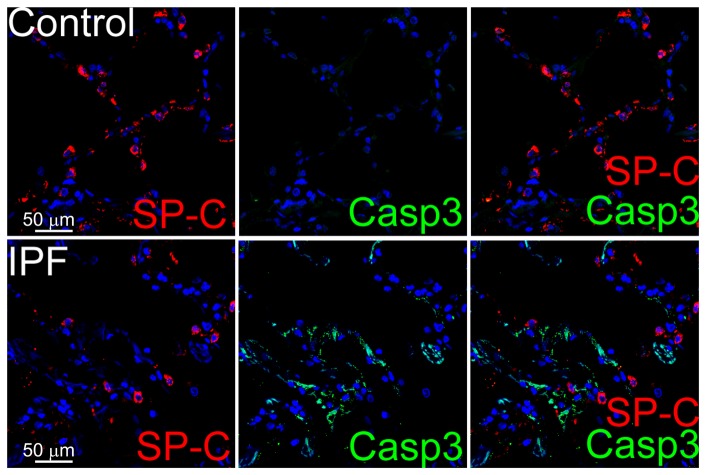
The lack of MMP-8 staining in type II alveolar epithelial cells in IPF lungs is not due to increased rates of apoptosis of these cells. Lung sections from a control and IPF patient were stained with a red fluorophore for a marker of type II alveolar epithelial cells (SP-C, left column) and with a green fluorophore for active caspase-3 (Casp3; middle column as a marker of apoptotic cells). Merged images (right) show apoptotic cells (stained green) in an area of severe fibrosis in the IPF lung, but these apoptotic cells are not ATII cells as assessed by the lack of co-localization of active caspase-3 and SP-C staining. There is no apoptosis in alveolar epithelial cells in the control lung, as expected. The images are representative of immuno-stained lung sections from 2 control subjects and 3 patients with IPF. Magnification is X 600.

To determine whether the reduced MMP-8 staining in ATII cells in IPF lungs is linked to increased rates of ATII cell apoptosis which has been reported in IPF lungs [28], [29], we double immunostained IPF versus normal lungs for a marker of apoptosis (active caspase-3) and a marker of ATII cells (surfactant protein C; SP-C). Positive immunostaining for active caspase-3 is present in areas of moderately severe and severe fibrosis in IPF lung parenchyma but not in normal lung parenchyma (Fig. 6). However, the apoptotic cells in IPF lung parenchyma are not ATII cells as there is minimal or no co-localization of the staining for active caspase-3 and SP-C.

### MMP-8 levels in plasma samples

**Figure 7:**
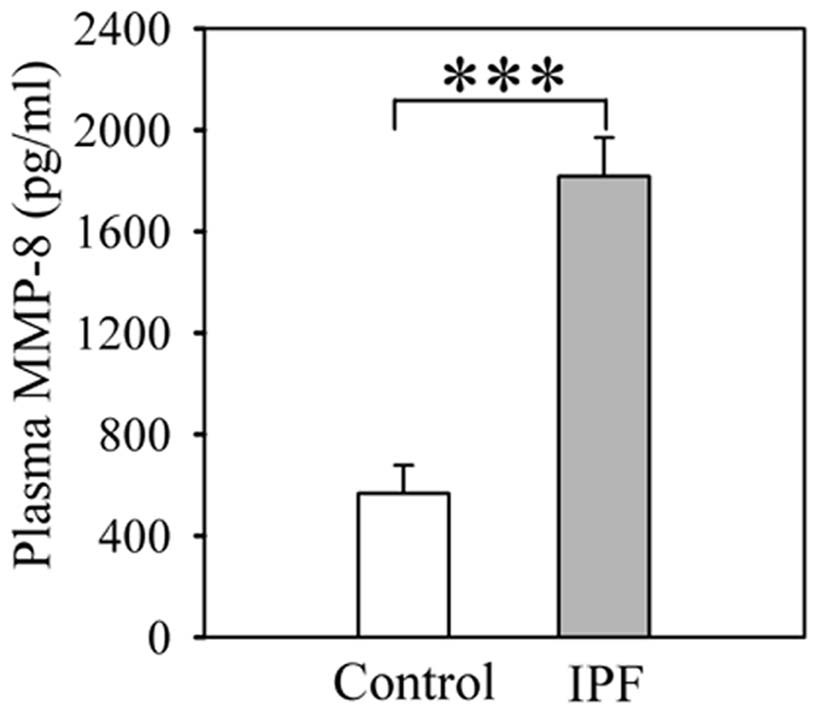
MMP-8 levels are increased in IPF plasma samples. Plasma samples were obtained from patients with IPF and healthy volunteers (control). MMP-8 protein levels were measured using an ELISA. Data are mean + SEM; n = 21 healthy volunteers and n = 73 patients with IPF; asterisks indicate p<0.001.

Plasma MMP-8 protein levels are >3–fold higher in IPF patients when compared with levels in healthy volunteers (Fig. 7). Table 1 shows the demographic data on these subjects.

**Table 1:** Demographic data on subjects recruited to measure MMP-8 levels in blood samples.

	IPF patients	Control subjects¶	P value
Number of subjects	73	25	
Age (years)	68.7 (7.9)†	62.9 (10.4)	0.006
Gender (% male)	69.2	80.0	0.446
Caucasian (%)	96.8	96.0	0.642
Hispanic (%)	3.2	12.0	0.276
FVC (L)	2.65 (0.8)	N/A††	
FVC (% predicted)	69.8 (18.3)	N/A	
TLC (L)	4.0 (1.1)	N/A	
TLC (% predicted)	67.5 (21.0)	N/A	
DLCO (mL/min/mmHg)	13.3 (6.1)	N/A	
DLCO (% predicted)	49.9 (18.7)	N/A	
Smoking (pack years)	23.3 (14.1)	2.08 (8.2)	<0.001

† Data shown are mean results (with SD in parentheses) in both groups for age, gender, forced vital capacity (FVC), FVC % predicted (% pred), total lung capacity (TLC), TLC % predicted, diffusing capacity of the lung for carbon monoxide (DLCO), DLCO % predicted, and smoking pack years.

†† N/A: not available.

¶ MMP-8 levels were measured in plasma samples from 21 of the 25 control subjects, MMP-8 levels were measured in leukocytes isolated from up to 7 of the 25 control subjects, and MMP-8 levels were measured in both plasma and leukocyte samples obtained from 3 of the 25 control subjects.

### MMP-8 levels in blood neutrophils

**Figure 8:**
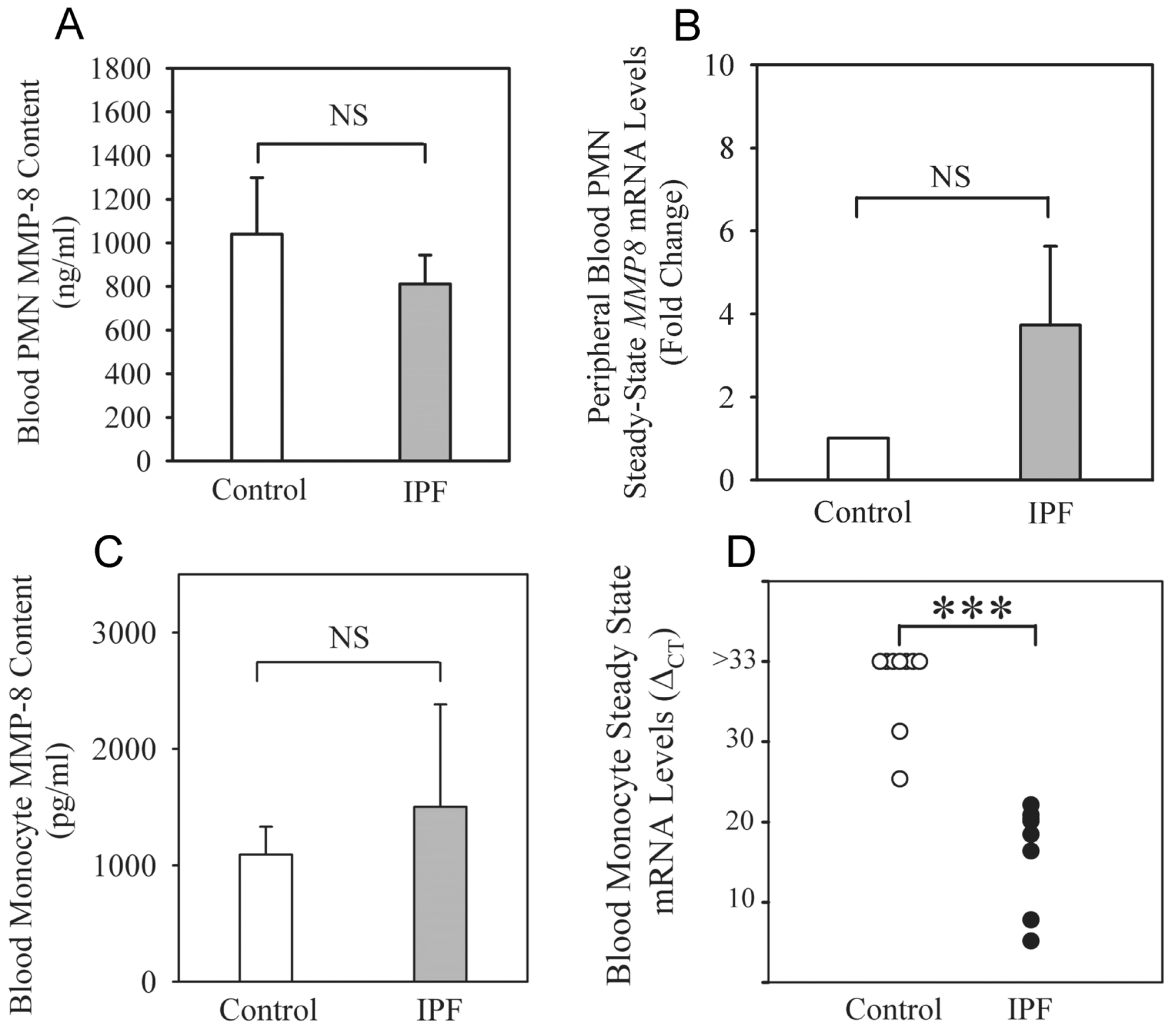
MMP-8 expression is increased in peripheral blood monocytes but not in peripheral blood neutrophils. Neutrophils and monocytes were isolated from blood samples obtained from IPF patients being evaluated for lung transplantation and healthy volunteers. MMP-8 protein levels were measured in neutrophil lysates (**A**) and monocyte lysates (**C**) (all prepared at 5×10^6^ cells/ml) using an ELISA. In **A** and **C**, data are mean **+** SEM, n = 6–7 healthy volunteers and n = 4–22 IPF patients. MMP-8 steady-state mRNA levels were measured in RNA isolated from neutrophils (**B**) and monocytes (**D**) obtained from 7–9 control subjects and 6–9 IPF patients using a commercial gene expression assay for MMP-8 and 18 S as an endogenous reference. Data are expressed as fold change + SEM in **B** and as ΔC_T_ in **D** (C_T_ for MMP-8 - C_T_ for 18 S as MMP-8 transcripts were not detected in 7 out of 9 controls [C_T_ >60 for MMP-8 and plotted as ΔC_T_ >33] or expressed at low levels in monocytes from 2 of the control subjects). Asterisks indicate p<0.001.

As there is evidence that blood neutrophils are activated in IPF patients [23], we hypothesized that MMP-8 protein levels are lower in extracts of blood neutrophils but higher on the surface of blood neutrophils from IPF patients compared with levels in control subjects. However, we show that MMP-8 protein levels are similar in blood neutrophil extracts from IPF patients and control subjects (Fig. 8A). The percentage of neutrophils that stain positively for surface MMP-8 (which increases when neutrophils are activated [12], [17]) is also similar in IPF patients and controls (17.8± SEM 5.6%; n = 32 versus 3.4±1.9%; n = 5, respectively; p = 0.79). MMP-8 is not thought to be regulated at the steady state mRNA level in blood PMNs. However, we detected MMP-8 transcripts in PMNs from healthy donors using qRT-RT-PCR. IPF patients and controls have similar low levels of MMP-8 mRNA transcripts in blood neutrophils (Fig. 8B).

### MMP-8 levels in blood monocytes

In healthy donors, blood monocyte extracts contain less MMP-8 (∼1 ng/5 million cells) than neutrophil extracts (∼1000 ng/5 million cells) as expected (Figs. 8C and 8A, respectively). MMP-8 protein levels are similar in blood monocyte extracts from IPF patients and control subjects (Fig. 8C). In blood monocytes, MMP-8 mRNA levels are very low or not detectable in normal volunteers (cycle threshold [C_T_] >60 cycles for 7 out of 9 healthy volunteers and ≥25.3 in 2 healthy volunteers) whereas the C_T_ for the IPF patients ranges from 5.16 to 22.11. Thus, it is not possible to calculate fold change in monocyte MMP-8 steady state mRNA levels for IPF patents versus healthy subjects using the ΔΔC_T_ method. Instead, we report MMP-8 steady state mRNA levels using the ΔC_T_ method for IPF patients versus controls (C_T_ for MMP-8 - C_T_ for 18 S as the housekeeping gene). The lower ΔC_T_ for IPF patients indicates higher MMP-8 mRNA levels in monocytes from IPF patients compared with control subjects (Fig. 8D). We used publicly-available microarray gene expression databases to compare MMP-8 expression in peripheral blood mononuclear cells (PBMCs) from COPD versus healthy control subjects [30] and sarcoidosis patients versus healthy control subjects [31]. Our analysis shows that MMP-8 transcripts are not detected in COPD PBMCs and MMP-8 expression is not significantly increased in PBMCs from patients with sarcoidosis (Table S2).

### BALF MIP-1α and IP-10 levels

MMP-8 reduces lung levels of MIP-1α and IP-10 in bleomycin-treated mice to promote pulmonary fibrosis [10]–[12]. However, BALF levels of MIP-1α and IP-10 are not significantly different in IPF patients and control subjects, and there are trends towards higher (rather than lower) levels of both mediators in IPF patients (Table S3). Among the IPF patients, BALF MMP-8 levels do not correlate significantly with BALF levels of either MIP-1α or IP-10 (Spearman Rank Correlation Coefficients  =  0.07 [p = 0.84] and 0.31 [p = 0.29], respectively).

### MMP-8 levels in plasma, BALF, and PBMCs and clinical outcomes

**Figure 9:**
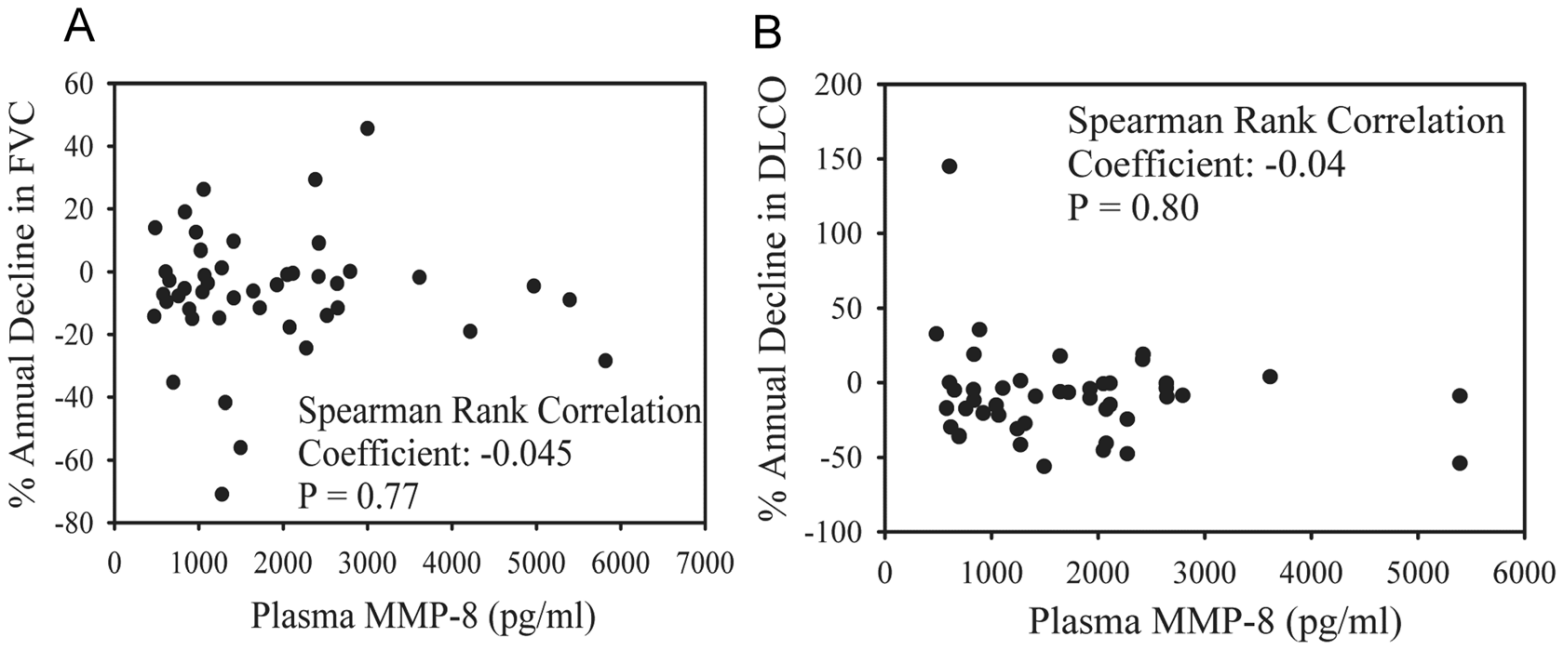
MMP-8 levels in plasma do not correlate with decline in pulmonary function in IPF patients. Serial FVC and DLCO measurements of lung function were performed on IPF patients being evaluated for lung transplantation. Annual rates of decline in both measures were calculated as described in Methods and plotted against plasma MMP-8 levels measured using an ELISA. Correlations between MMP-8 plasma levels and absolute annual rate of decline in FVC (**A**) or DLCO (**B**) were calculated using the Spearman Rank Correlation Coefficient; 45 IPF patients were studied in **A** and **B**.

MMP-8 plasma levels do not significantly correlate with the annual absolute rate of decline in FVC (Fig. 9A) or DLCO (Fig. 9B) expressed as a percentage of the patients' baseline FVC or DLCO, or absolute FVC and DLCO (data not shown). In addition, plasma MMP-8 levels do not correlate with initial FVC or DLCO at presentation (data not shown). Plasma MMP-8 levels in 66 IPF patients also do not correlate with mortality as assessed using the Cox proportional-hazards model analysis (data not shown).

MMP-8 protein levels in BALF from IPF patients (n = 32) do not correlate with annual absolute rate of decline in FVC or DLCO (data not shown). An analysis of a publicly-available PBMC microarray gene expression dataset on IPF patients [32] for PBMC MMP-8 steady state mRNA levels shows that these levels do not correlate with mortality (personal communication, Naftali Kaminski, MD, Yale University School of Medicine).

## Discussion

MMP-8 has been identified as a pro-fibrotic proteinase in the lungs of bleomycin-treated mice [10], [11] but much less is known about its roles in human IPF. Herein, we report that MMP-8 plasma levels are higher in IPF patients compared with control subjects in agreement with results reported in one prior study [18]. However, we add to this literature by showing that MMP-8 plasma levels do not correlate with annual rate of decline in lung function or mortality in IPF patients. We hypothesized that MMP-8 levels in blood neutrophils would be altered in IPF patients, as MMP-8 is most highly expressed by neutrophils, and another study reported that blood neutrophils are activated in IPF patients as indicated by increased plasma neutrophil elastase levels in IPF patients [23]. Surprisingly, we found no differences in total cellular or surface levels of MMP-8 in/on blood neutrophils from IPF patients when compared with cellular levels in control subjects. We report for the first time that MMP-8 gene expression levels are robustly upregulated in blood monocytes in IPF patients, but MMP-8 gene expression in PBMCs does not correlate with mortality in IPF patients when a publicly-available microarray dataset is analyzed.

Consistent with results from prior studies [18], [20], [21], MMP-8 protein levels are increased in BALF samples from our IPF cohort but do not correlate with pulmonary function. Thus, BALF MMP-8 levels are unlikely to serve as a useful prognostic biomarker for IPF. However, our results add to the literature by showing that the main form of MMP-8 that is increased in IPF BALF is active MMP-8. Although we expected that neutrophils and fibroblasts would express MMP-8 in IPF lungs, our study shows for the first time that lung macrophages and bronchial epithelial cells are the main sources of MMP-8 in IPF lungs. MMP-8 expressed by macrophages and bronchial epithelial cells may contribute to the fibrotic process in IPF lungs.

Until now, MMP-8 has not been well studied in blood samples, which are easier to obtain than lung samples for measuring biomarkers. Although mean plasma MMP-8 levels are >3-fold higher in IPF patients compared with controls, our results indicate plasma MMP-8 levels are unlikely to serve as a useful prognostic biomarker for IPF patients as they do not correlate with rate of decline in lung function or mortality in IPF patients. Additionally, plasma MMP-8 levels are not specific for IPF as increased plasma MMP-8 levels occur in patients with active coronary artery disease [33] and breast cancer [34]. However, additional studies are needed to determine whether plasma MMP-8 levels correlate with other clinical outcomes and parameters in IPF patients including disease exacerbations.

Blood neutrophil MMP-8 protein and steady state mRNA levels are similar in IPF patients and control subjects. Until now, it has been thought that MMP-8 is not synthesized de novo by circulating mature neutrophils. Rather, MMP-8 is synthesized by bone marrow precursors of neutrophils and pre-formed MMP-8 protein is stored in neutrophil specific granules and released from these granules into the extracellular space when the cells degranulate [35]. However, in the current study, we detected MMP-8 mRNA transcripts in neutrophils from both IPF patients and healthy control subjects and this likely reflects the more sensitive qRT-RT-PCR method used herein when compared with methods used previously [36]. Membrane-bound MMP-8 on murine PMNs contributes significantly to MMP-8's anti-inflammatory activities in mice with ALI [12], [17]. However, we found no differences in the expression of membrane-bound MMP-8 on PMNs from IPF patients versus controls indicating that this form of the proteinase is unlikely to contribute to lung fibrosis in human IPF patients.

MMP-8 is not thought to be a monocyte product. However, we detected MMP-8 mRNA transcripts in monocytes from some healthy subjects, and MMP-8 gene expression is significantly increased in monocytes from IPF patients. The reasons for this finding are not clear, but as MMP-8 gene expression increases in macrophages activated in vitro, mediators released in IPF lungs may induce MMP-8 expression in monocytes. Although MMP-8 gene expression is increased in IPF monocytes, we detected similar low levels of MMP-8 protein in extracts of blood monocyte from both healthy subjects and IPF patients. Likely, monocytes synthesize and rapidly release (rather than store) MMP-8 protein. It is noteworthy that gene expression profiles of PBMCs (lymphocytes and monocytes) have recently been shown to predict poor outcomes in IPF patients [32]. However, MMP-8 gene expression levels in PBMCs do not correlate with mortality in IPF patients in this publicly-available dataset (personal communication, Naftali Kaminski, MD). Other studies report that patients with COPD and sarcoidosis have increased MMP-8 gene expression in PBMCs [30], [31], but we were not able to confirm these findings when we analyzed other publicly-available microarray gene expression datasets of PBMCs from patients with sarcoidosis or COPD versus healthy control subjects (see Table S2). However, increased MMP-8 gene expression in blood monocytes is unlikely to be a predictive or prognostic biomarker for IPF.

Although BALF levels of MMP-8 have been reported to be elevated in IPF patients previously [18], [20], [21], until now the crucial cellular sources of pro-fibrotic MMP-8 in the lung have not been identified. We report for the first time that macrophages are one key cell type contributing to the elevated MMP-8 levels in IPF lungs, and macrophages in areas of mild as well as severe fibrosis robustly express MMP-8. While bronchial epithelial cells in control lungs do not express MMP-8, robust staining for MMP-8 is detected in bronchial epithelial cells in moderately severe and severe areas of fibrosis in IPF lungs. MMP-8 is also expressed by bronchial epithelium and macrophages in patients with bronchiectasis [37]. Thus, under pathologic conditions, mediators released in the lung may induce MMP-8 expression by bronchial epithelial cells and lung macrophages. Whether MMP-8 expressed by bronchial airway epithelium contributes to the fibrotic process in IPF lungs is not clear. However, MMP-8 expressed by distal airway epithelium could contribute to epithelial to mesenchymal transition.

We detected MMP-8 staining in ATII cells in our control lungs, which has not been reported previously. However, AT II cells have minimal or no MMP-8 expression in areas of moderately severe and severe fibrosis in IPF lungs. Although other studies report that ATII cells have increased apoptosis rates [28], [29], our immunostaining results demonstrate apoptosis in cells other than ATII cells in IPF lungs (possibly ATI cells). Thus, it is unlikely that the reduced MMP-8 staining in ATII cells in IPF lungs is due to reduced viability of these cells. While activated murine lung fibroblasts express MMP-8 [10], we did not detect expression of MMP-8 by IPF lung fibroblasts. This underscores the importance of confirming findings obtained in animal models in samples from humans with the disease.

A recent study reported that MMP-8 protein levels are not increased in IPF lung tissue when compared with donor lungs that were not used for lung transplantation as assessed by western blotting [19]. In contrast, we found that MMP-8 protein levels are increased in homogenates of lung samples. The reasons for these different results are not clear but could be related to the status of the donor lung tissue studied. If the discarded donor lung tissue was obtained from donors having lung inflammation or injury due to mechanical ventilation, infection, or other inflammatory responses in the study of Nkyimbeng et al. [19], this could have elevated MMP-8 protein levels in the donor lung and contributed to the lack of a difference in MMP-8 levels between IPF and donor lungs in this study. It is noteworthy that Nkyimbeng et al. detected strong positive staining for MMP-8 in alveolar septae of their control lungs whereas we detected MMP-8 staining only in ATII cells in control lung sections. In addition, different antibodies were used in our study versus that of Nkyimbeng et al. which could recognize different epitopes in MMP-8 leading to differences in the results if these epitopes undergo different proteolytic processing in tissues.

MMP-8 promotes lung fibrosis in mice by decreasing lung levels of MIP-1α and IP-10 [10]. However, in our study, MMP-8 BALF levels do not correlate indirectly with BALF levels of MIP-1α or IP-10 in IPF patients, suggesting that these chemokines may not be MMP-8 substrates and/or MMP-8 has other pro-fibrotic activities in IPF lungs. Also, measuring levels of MMP-8 and chemokines in BALF may not reflect proteolytic events occurring in lung microenvironments other than the alveolar space. It is also noteworthy that delivering a truncated form of MMP-8 to the livers of rats with hepatic cirrhosis reduces hepatic fibrosis [38]. Furthermore, MMP-8 has protective anti-inflammatory activities in mice with acute lung injury [12] and allergen-induced airway inflammation [39], but pro-inflammatory activities during acute hepatic injury in mice [40]. Thus, more studies are needed on human cells and samples to determine whether MMP-8 has pro-fibrotic or beneficial activities in the human lung.

Our study has some limitations including the relatively small number of subjects studied. Also, our healthy subjects are modestly but significantly younger than our IPF patients. MMP-8 levels increase in tissues in mice as they age [41]. However, our analysis of MMP-8 plasma levels in older healthy controls (≥60 years of age; n = 14) versus younger health controls (<60 years of age; n = 11) revealed similar median MMP-8 plasma levels (364 vs. 645 pg/ml, respectively; p = 0.119). Thus, it is unlikely that the higher MMP-8 plasma levels in IPF patients are due to their greater age, but this needs to be confirmed in future studies.

Most of our IPF patients have a history of smoking cigarettes while most of our control subjects are non-smokers. Smoking status could affect MMP-8 levels in blood and lung samples as human PMNs incubated with cigarette smoke extract release increased amounts of MMP-8 and MMP-9 [42]. Additionally, plasma MMP-8 levels correlate with smoking status [43]. Therefore, the greater cigarette smoking history in our IPF cohort compared with that in our control subjects could potentially contribute to the higher plasma MMP-8 levels observed in the IPF patients.

In conclusion, we confirm that MMP-8 levels are robustly increased in plasma samples from IPF patients. However, we report for the first time that MMP-8 plasma levels do not correlate with mortality or decline in lung function in IPF patients. We identify novel cellular culprits expressing increased levels of MMP-8 in IPF patients (blood monocytes, lung macrophages, and bronchial epithelial cells). We also provide new insights into the form of MMP-8 that is increased in IPF lungs (active MMP-8). Additional studies are needed to determine whether plasma MMP-8 levels correlate with other clinical parameters not studied herein. If MMP-8 is found to have pro-fibrotic activities in human as well as murine lungs, our results may help guide future studies testing the efficacy of novel therapies targeting MMP-8 in halting the progression of lung fibrosis in IPF patients.

## Supporting Information

Table S1 **Demographic data on subjects recruited to measure MMP-8 protein levels or forms in BALF samples.** ^†^Data are expressed as mean values for both groups (with SD in parentheses) for age, gender, total lung capacity (TLC), and diffusing capacity of the lung for carbon monoxide (DLCO). ^††^BALF from 7 of the 12 control subjects was used to measure MMP-8 protein levels using an ELISA and BALF from 9 of the 12 control subjects was subjected to Western blotting to quantify MMP-8 forms.
(DOC)

Table S2 **Results of analysis of Gene Expression Omnibus (GEO) publicly-available microarray gene expression databases for PBMCs in the National Center for Biotechnology Information (NCBI).** ^†^Gene expression datasets on peripheral blood mononuclear cells that are publicly-available *(* http://www.ncbi.nlm.nih.gov/geo/ *)* were analyzed using the GEO2R interactive web tool and the GEO query and limma R packages [44] from the Bioconductor project. ^††^P-value after adjustment for multiple testing using the Benjamin and Hochberg test [45].
(DOC)

Table S3 **MIP-1α and IP-10 levels in BALF samples from IPF cases and control subjects.** ^†^MIP-1α and IP-10 were measured in BALF samples from 8 IPF cases and 5 control subjects using ELISA kits. ^‡^Results are expressed as mean (SEM) values.
(DOC)
