# Supplementary material for: Mononuclear Phagocytes and Airway Epithelial Cells: Novel Sources of Matrix Metalloproteinase-8 (MMP-8) in Patients with Idiopathic Pulmonary Fibrosis
Source: PLoS One. 2014 May 14;9(5):e97485. doi: 10.1371/journal.pone.0097485 (PMC4020836; doi:10.1371/journal.pone.0097485)
Supplement: Table S1 — Demographic data on subjects recruited to measure MMP-8 protein levels or forms in BALF samples. †Data are expressed as mean values for both groups (with SD in parentheses) for age, gender, total lung capacity (TLC), and diffusing capacity of the lung for carbon monoxide (DLCO). ††BALF from 7 of the 12 control subjects was used to measure MMP-8 protein levels using an ELISA and BALF from 9 of the 12 control subjects was subjected to Western blotting to quantify MMP-8 forms. (DOC) [file pone.0097485.s001.doc]

|  | IPF patients | Control subjects^††^ | P value |
| --- | --- | --- | --- |
| Number of subjects | 32 | 12 |  |
| Age (years) | 64.3 (7.7)^†^ | 50.8 (15.8) | 0.013 |
| Gender (% male) | 71.0 | 63.6 | 0.939 |
| TLC (% predicted) | 70.4 (17.5) | 100.51 (10.0) | < 0.001 |
| DLCO (% predicted) | 51.1 (19.4) | 112.6 (9.6) | < 0.001 |
