# Supplementary material for: Mononuclear Phagocytes and Airway Epithelial Cells: Novel Sources of Matrix Metalloproteinase-8 (MMP-8) in Patients with Idiopathic Pulmonary Fibrosis
Source: PLoS One. 2014 May 14;9(5):e97485. doi: 10.1371/journal.pone.0097485 (PMC4020836; doi:10.1371/journal.pone.0097485)
Supplement: Table S2 — Results of analysis of Gene Expression Omnibus (GEO) publicly-available microarray gene expression databases for PBMCs in the National Center for Biotechnology Information (NCBI). †Gene expression datasets on peripheral blood mononuclear cells that are publicly-available ( http://www.ncbi.nlm.nih.gov/geo/ ) were analyzed using the GEO2R interactive web tool and the GEO query and limma R packages [44] from the Bioconductor project. ††P-value after adjustment for multiple testing using the Benjamin and Hochberg test [45]. (DOC) [file pone.0097485.s002.doc]

| NCBI Geoset number^†^ | Populations studied | Platform used | Fold change in MMP-8 expression | Adjusted P-value^††^ |
| --- | --- | --- | --- | --- |
| ***GSE42057*** | 95 COPD patients versus 41 healthy controls [30] | [HG-U133_Plus_2] Affymetrix Human Genome U133 Plus 2.0 Array | MMP-8 transcripts not detected | ---- |
| ***GSE37912*** | 35 patients with sarcoidosis versus 39 healthy controls [31] | [HuEx-1_0-st] Affymetrix Human Exon 1.0 ST Array [transcript (gene) version] | 1.68 | 0.062 |
