# Supplementary material for: Mononuclear Phagocytes and Airway Epithelial Cells: Novel Sources of Matrix Metalloproteinase-8 (MMP-8) in Patients with Idiopathic Pulmonary Fibrosis
Source: PLoS One. 2014 May 14;9(5):e97485. doi: 10.1371/journal.pone.0097485 (PMC4020836; doi:10.1371/journal.pone.0097485)
Supplement: Table S3 — MIP-1α and IP-10 levels in BALF samples from IPF cases and control subjects. †MIP-1α and IP-10 were measured in BALF samples from 8 IPF cases and 5 control subjects using ELISA kits. ‡Results are expressed as mean (SEM) values. (DOC) [file pone.0097485.s003.doc]

|  | IPF patients | Control subjects | P value |
| --- | --- | --- | --- |
| BALF MIP-1α^†^ | 5.6 (2.4)^‡^ pg/ml | 1.9 (0.9) pg/ml | 0.171 |
| BALF IP-10 | 33.8 (10.9) pg/ml | 8.1 (2.9) pg/ml | 0.265 |
